# A Unique Finite Element Modeling of the Periodic Wave Transformation over Sloping and Barred Beaches by Beji and Nadaoka's Extended Boussinesq Equations

**DOI:** 10.1155/2013/306535

**Published:** 2013-06-17

**Authors:** Mohammad Hadi Jabbari, Parviz Ghadimi, Mesbah Sayehbani, Arsham Reisinezhad

**Affiliations:** Department of Marine Technology, Amirkabir University of Technology, P.O. Box 15875-4413, Tehran, Iran

## Abstract

This paper presents a numerical model based on one-dimensional Beji and Nadaoka's Extended Boussinesq equations for simulation of periodic wave shoaling and its decomposition over morphological beaches. A unique Galerkin finite element and Adams-Bashforth-Moulton predictor-corrector methods are employed for spatial and temporal discretization, respectively. For direct application of linear finite element method in spatial discretization, an auxiliary variable is hereby introduced, and a particular numerical scheme is offered to rewrite the equations in lower-order form. Stability of the suggested numerical method is also analyzed. Subsequently, in order to display the ability of the presented model, four different test cases are considered. In these test cases, dispersive and nonlinearity effects of the periodic waves over sloping beaches and barred beaches, which are the common coastal profiles, are investigated. Outputs are compared with other existing numerical and experimental data. Finally, it is concluded that the current model can be further developed to model any morphological development of coastal profiles.

## 1. Introductions

Coastal profile can vary considerably during a year or even during a single storm event. As a result of this change in profile, long shore bars can be formed and existing bars can be shifted over barred beaches or destroyed and constant slope beaches are built. The cross-shore sediment transport plays a significant role in the development of the coastal profiles. In this case, long shore bars will be formed on a sandy constant slope profile as a result of the interaction between the onshore and offshore transport flows in the nearshore zone [1]. In the meantime, the behavior of periodic waves over the sloped and barred beaches is different. The nonlinearity of the periodic wave travelling over beaches with constant slope profile continually increases following the same trend over the barred beaches. However, in the case of waves travelling over the barred beaches, after the waves pass over the bars they get decomposed and their nonlinearity decreases. The breaking and nonbreaking conditions may occur in each of the mentioned cases, and studying them may be significant in development of the coastal profile.

Several governing equations exist for simulation of waves over the coastal zone. Fully nonlinear potential flow (FNPF) and incompressible Navier-Stokes equation are common governing equations that are used for simulation of the periodic wave interactions in the coastal zone. While two- and three-dimensional Navier-Stokes equations provide accurate mathematical models for wave motions, the numerical analysis of such an actual problem would involve a huge computational domain with an unknown water surface elevation. Therefore, as an alternative, the popular shallow water equations are used to simulate the periodic wave interactions which have been developed rapidly in the last decades. By integrating the Navier-Stokes equation over depth, the three-dimensional and two-dimensional problems can be reduced to two-dimensional and one-dimensional computational problems, respectively. Shallow water equations substantially reduce the computational cost associated with the wave modeling when diffraction, refraction, shoaling, and harmonic interaction are of particular importance. Boussinesq equation is one type of shallow water equations which is derived from the Euler or Navier-Stokes equations. In deriving the Boussinesq equations, free surface of the water and the averaged velocities are approximated by solving the continuity and momentum equations, respectively. These equations are ordinarily applied for determining the water surface elevation inside the harbors and wave interactions in near the shore. 

The improvement of linearized dispersion property of the Boussinesq equations has been the basis for all developments of these types of equations from the past to the current time. Any improvement in the dispersion characteristic of these equations would bring about a better accuracy in results associated with the deeper waters. Validity of the linearized dispersion property of the original Boussinesq equations [2, 3] is limited to actual shallow waters, that is, for the range of *kh* ≤ 0.76 (*k* is the wave number and *h* is the water depth). With derivation of the developed Boussinesq equations [4–6], linearized dispersion property of the Boussinesq equations has drastically improved and they can even be applied to the relatively deep water, that is, for the range of *kh* ≤ 3. However, in recent years, by derivation of high-order Boussinesq equations [7–10], the applicable range of the Boussinesq equations have increased (6 ≤ kh ≤ 40), but nonetheless, the complexities involved in sophisticated mathematical form of high-order equations has made the numerical modeling and/or finding the accurate numerical solution very difficult. 

In the present work, the one-dimensional Beji and Nadaoka extended Boussinesq equations [6] have been used as governing equations to model regular wave shoaling and its decomposition in nearshore zone. Needless to say that, at nearshore zone, the dispersive properties of the extended Boussinesq equations and high-order Boussinesq equations are the same. The Beji and Nadaoka extended Boussinesq equations (BNBE) is derived by a simple mathematical manipulation of Peregrine's equations. 

From the mathematical viewpoint, the system of BNBE contains third-order free surface derivatives in momentum equations, and also the form of BNBE is not so straightforward when employed for variable depth. For this reason, the BNBE have not been considered as frequent as the other equations such as Nwogu Boussinesq equations by the researchers. Furthermore, finite difference scheme has been applied in most of the numerical models of the time-dependent Boussinesq-type equations [3, 6, 11]. Although, the usage of finite difference approach is easy, the finite element method is more efficient. While Finite element method can be applied to discretize complex domain, the finite difference method does not have the capability of handling the irregular boundaries which are encountered in several coastal engineering problems. Another important characteristic of the finite element scheme is its superiority in applying a finer mesh for some regions of interest, which would indeed yield in the minimization of the number of required grid points. Therefore, one can state with relative certainty that the use of finite element method for solving Boussinesq-type equations is preferred over the finite difference method by researchers. For example, Do Carmo et al. [12] and Do Carmo et al. [13] presented numerical solutions for the Boussinesq equations based on finite element method, excluding the dispersion modification terms. Ghadimi et al. [14] applied Galerkin finite element method for solving the extended Boussinesq equations and calculated the solitary wave shoaling on plane beaches. Li et al. [15] applied BNBE for simulation of regular and solitary wave's dispersion in nearshore zone. Their numerical method was based on a finite elements approach with special treatment of third-order spatial derivatives. 

The current paper presents a new and particular technique based on finite element scheme to investigate the propagation of wave over sloping and barred beaches, where both nonlinearity and dispersion properties of the waves are accurately modeled. Finite element method used here is based on the rewritten equations in lower-order differentials form by introducing an auxiliary variable [15, 16]. The auxiliary variable is derived by introducing a novel form of related equations. By showing the good modeling ability of the wave properties, it is also demonstrated how capable the suggested scheme can be for modeling the morphological development. Accordingly, the most typical beach profiles such as sloping beaches and barred beaches are considered in this study. 

## 2. Boussinesq Equations System

The physical system depicting the entire seabed and wave profile can be represented by a water depth *H*, a wavelength *λ*, and a wave amplitude *a*. The nonlinearity and dispersion existing in the system are denoted by the ratios *ϵ* and *σ* as follows:

(1) ϵ=aH,σ=Hλ.

As discussed in the literature, BNBE are produced by adding extra dispersive terms to Peregrine's momentum equations [2]. Mathematical form of these equations in one-dimensional domain is as follows:

(2) ηt+∂∂x(Hu)=0,

(3) ut+u∂u∂x+g∂η∂x=(1+β)h[h3∂2ut∂x2+∂h∂x∂ut∂x]+βgh[h3∂3η∂x3+∂h∂x∂2η∂x2],

where *β* is a free parameter and *u*(*x*, *t*) is the depth-averaged velocity vector in BNBE system.

The value of free parameters in extended boussinesq equation systems are obtained from the best agreement achieved between the linearized and exact dispersion relations. After introducing a new boussinesq equations system, the equations are linearized and phase velocities (*C*) or group velocities will be compared to the exact dispersion relation or Stokes' first-order theory (*C* ^2^ = *gh*(tanh⁡*kh*)/(*kh*)). The linearized dispersion relation properties of the BNBE that can be found [6] are given as in

(4) C2=gh[(1+β(kh)2/3)1+(1+β)(kh)2/3].

In this paper, the value of *β* = 0.177 has been considered. This value is the best choice because the best agreement between the linearized dispersion relations (4) and the exact dispersion relations is achieved at this value, with a maximum error of less than 6%.

## 3. Numerical Method

### 3.1. Finite Element Formulation

Application of a linear finite element method from the view point of simplicity in formulation is similar to the application of a central finite difference method, but with this difference the finite element can also be applied to the unstructured mesh. Because of the fact that (3) includes a third-order derivative in space, we cannot directly apply the linear Galerkin finite element scheme to the system of equations. Linear elements can only be applied, when the weighted residual form of the problem contains second-order spatial gradients (second-order free surface derivatives in BNBE) in which case the linear finite element approximation cannot be applied. Therefore, special treatment of the second-order derivatives becomes necessary. 

Walkley and Berzins [16] presented a new technique, based on the linear Galerkin finite element method, to solve the Nwogu Boussinesq equations (NBE). To overcome the difficulty associated with the third-order spatial derivatives in NBE, they introduced an auxiliary variable and rewrote equations system in its lower-order form. Applying this auxiliary equation, the high-order terms of Nwogu Boussinesq equations are eliminated and thus the solution of NBE by linear elements becomes possible. The form of the suggested auxiliary variable is based on the nature of the differential form of NBE. Therefore, this model is only limited to special cases where this auxiliary term is inherently embodied in the governing equations like NBE, and not equations like BNBE (e.g., [6]) that do not explicitly possess such a term. 

However, in this study, a flexible method for choosing the auxiliary variable is introduced which is not restricted by the mathematical form of the governing equations. For spatially discretizing the equations, the standard Galerkin finite element scheme is utilized. The finite element formulation of the dependent variables is as follows:

(5) f≈∑i=1ndNjfi,

where *f*_*i*_, *N*_*j*_, and *nd* are the values of any independent variable at the nodal points, the standard basis functions, and the number of nodes, respectively. To discretize the spatial domain *Ω*, nonoverlapping elements are applied to subdivide the continuum and subsequently each element and node is numbered. The presence of third-order spatial derivatives in momentum equation (3) makes the application of a standard linear interpolation finite element method for conversion of the integral equation to allegorical equations difficult. A particular scheme for overcoming this difficulty is the usage of an auxiliary equation that reduces the order of the original equation. Based on this scheme, an extra equation for *w* is introduced in the Boussinesq equations system as follows:

(6) ηt+∂∂x(Hu)=0,

(7) ut+u∂u∂x+g∂η∂x=(1+β)h[h3∂2ut∂x2+hx∂ut∂x]+∂w∂x,

(8) w=βgh[h3∂2η∂x2+hx∂η∂x].

The basis of this auxiliary equation is the fact that all nodal values over their corresponding elements are assumed constant. Multiplying these equations by the test functions *N*_*k*_ and integrating over the entire domain, the equations related to the Galerkin method are obtained. As a result of these steps, the continuity equation transforms to

(9) ∫Nk[ηt+∂∂x(Hu)]dΩe=0.

Pursuant to discretization of all terms in (7), the following matrix equation for an element *Ω* ^*e*^ is found:

(10) Mkiη˙i+QkijHiuj+QkijuiHj=0,

where the overdot in the first term represents the temporal differentiation with respect to time. Also, *M*_*ki*_ and *Q*_*kij*_ are defined as

(11) Mki=∫NkNidΩe,  Qkij=∫NkNi∂Nj∂xdΩe.

Using Green's theorem and applying the integration by parts, the momentum and auxiliary equations can also be written similar to (10) as in

(12) [Mki+(1+β)h(Eki−hxTki)]u˙i   +Qkijuiuj+gTkiηi−Tkiwi=0,

(13) Mkiwi=−βgh(Eki−hxTki)ηi,

where

(14) Tki=∫Nk∂Ni∂xdΩe,  Eki=13∫h∂Nk∂x∂Ni∂xdΩe−SAki.

*SA*_*ki*_ in (14) is the boundary integral that is defined as follows:

(15) SAki=13∫hNk∂Ni∂x·nxdΓe.

Here, Γ^*e*^ identifies the boundary of the element having unit normal *n*. Equations (14) and (15) become necessary only when natural boundary conditions are prescribed on portions of the boundaries of the computational domain. 

Finally, after discretization, the assembled global forms of (11) and (12) related to BNBE system take the form

(16) [M]{f˙}={E1}.

Also, the assembled global form of auxiliary equation (13) is obtained as follows:

(17) [M]{w}={E2}.

In (16) and (17), *M* is the coefficient matrix, *f* = *η*  or  *u*, while **E**_1_ and **E**_2_ are vectors that are determined by the known values of *u*,*η*, and *w*. System of linear equations in (17) can be solved explicitly at each time step, and (16) must be integrated in time by a typical high-order method.

### 3.2. Predictor-Corrector Scheme

If the variable *f* is discretized at *t* = *n*Δ*t*, the Adams-Bashforth-Moulton (ABM) method is adopted to integrate in time domain, in the following form [17].

(1) Right-hand side of (16) is evaluated at time levels *n*, *n* − 1, and *n* − 2.
(2) Equation (16) is temporally integrated by applying the predictor stage of the ABM method:
  (18) [M]{f}n+1=[M]{f}n+Δt12×[23{E1}n−16{E1}n−1+5{E1}n−2].
(3) Right-hand side of (16) is evaluated at time level *n* + 1.
(4) Equation (16) is temporally integrated by applying the corrector stage of the ABM method:
  (19) [M]{f}n+1=[M]{f}n+Δt24×[9{E1}n+1+9{E1}n−5{E1}n−1+{E1}n−2].

These steps are repeated until convergence is reached. In the present work, the system of equations is solved using generalized minimal residual (GMRES) method [18]. 

### 3.3. Boundary Conditions

 Inflow and outflow boundaries are two types of general boundary conditions which are employed here. 

#### 3.3.1. Inflow Boundary

For this type of boundary condition, initial value for water surface elevation for the entire computational domain is assumed zero, and the elevation *η* on the boundary for each time step is approximated as follows:

(20) η(x,t)=asin⁡(kx−ωt).

Linear theory [19] is applied to find the wave velocity for the incident wave elevation as in

(21) u=ωkhη.

The system of discretized equations becomes a complete set by addition of a specific variable of *w* on the inflow boundary for each model. Therefore, *w*_*i*_ for BNBE is determined as follows:

(22) wi−aβgh[−hk23sin⁡(kx−ωt)+khxcos⁡⁡(kx−ωt)]=0.

#### 3.3.2. Outflow Boundary

Minimization of the reflecting wave back into the domain is an important issue at the outflow boundary. Therefore, in this study, a viscous damping layer, that is, a sponge layer, is situated right before the outflow region to absorb the reflecting wave energy. For more detail of this method one can refer to the work done by Larsen and Dancy [20] and Li et al. [15].

### 3.4. Stability Analysis

In this segment, von Neumann stability analysis [21] has been conducted to investigate the stability of the suggested numerical model. For the stability analysis of BNBE, wave with small amplitude (linear) is considered over a constant water depth. Thus, (6), (7), and (8) are linearized as follows:

(23) η˙+h∂u∂x=0,u˙+g∂η∂x−(1+β)h23∂2u˙∂x2−∂w∂x=0,w−βgh23∂2η∂x2=0.

Matrix equations for each element *Ω* ^*e*^ can also be written as

(24) Mkiη˙i+hTkiuj=0,

(25) [Mki+(1+β)hEki]u˙i+gTkiηi−Tkiwi=0,

(26) Mkiwi+βghEkiηi=0.

Here, it is noted that

(27) ηin=η0GneIθi,

(28) uin=u0GneIθi,

(29) win=w0GneIθi,

where $I=\sqrt{-1}$ and *θ* = *k*Δ*x* are the imaginary unit and the phase angle, respectively. Also, (*η*_0_, *u*_0_) is the eigenvector of the problem, and *G* is the amplification factor. By substituting (24) into the Adams-Bashforth predictor method (18), we can obtain the following:

(30) Δx6(ηi−1n+1+4ηin+1+ηi+1n+1) =Δx6(ηi−1n+4ηin+ηi+1n)  −hΔt24[23(ui+1n−ui−1n)       −16(ui+1n−1−ui−1n−1)+5(ui+1n−2−ui−1n−2)].

Introducing (27) and (28) into (30) will result in

(31) β1G2(G−1)η0+β2(23G2−16G+5)u0=0,

where *β*_1_ and *β*_2_ are given as in

(32) β1=2cos⁡⁡θ+4,β2=hΔt4Δx(2sinθ)I.

Also, similar to the above scheme for (24), (25) becomes

(33) β3G2(G−1)u0+β4(23G2−16G+5)(gη0+w0)=0,

where *β*_3_ and *β*_4_ are given as in

(34) β3=(2cos⁡⁡θ+4)−2(1+β)h2(Δx)2(2cos⁡⁡θ−2),β4=Δt4Δx(2sinθ)I.

Expansion of (26) at every time step would lead to

(35) Δx6(wi−1n+4win+wi+1n)  −βgh23Δx(ηi−1n−2ηin+ηi+1n)=0.

By applying (27) and (29), it is concluded that

(36) w0=β5η0,

where

(37) β5=2βgh2(Δx)22cos⁡⁡θ−22cos⁡⁡θ+4.  

By introducing (36) to (33), (33) can be rewritten as

(38) β3G2(G−1)u0+β6(23G2−16G+5)η0=0,

where

(39) β6=β4(g−β5).

To have nontrivial solutions for eigenvector (*η*_0_, *u*_0_) for (31) and (38), we must have

(40) [G2(G−1)]2−β2β6β1β3[23G2−16G+5]2=0.

Equation (40) can be numerically solved for |*G*| in terms of *θ*, by assuming Δ*x* = 0.1 *h* and Cr = (0.5,1.0,1.5) where Cr is the Courant number. That is.

(41) Cr=gh(ΔtΔx).

**Figure 1:**
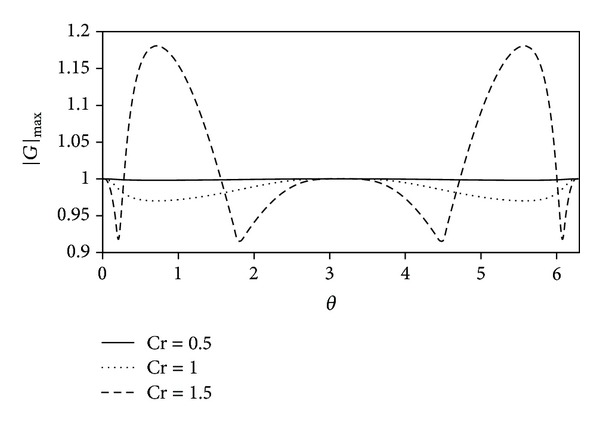
Largest modulus of the amplification factor |*G*|_max⁡_  in terms of phase angle *θ* for the predictor method.

Figure 1 demonstrates the largest modulus of the amplification factor in terms of *θ*. It is obvious that the predictor scheme is stable when the Courant number is smaller than or equal to unity.

The outlined technique for studying the stability of the Adams-Moulton predictor scheme in (42) can also be extended to examine the stability of the Adams-Moulton corrector method in (19). This would result in

(42) [G2(G−1)]2−α2α4α1α3[9G3+19G2−5G+1]2=0,

where

(43) α1=2cos⁡⁡θ+4,α2=hΔ8Δx(2sin⁡θ)I,α3=(2cos⁡⁡θ+4)−2(1+β)h2(Δx)2(2cos⁡⁡θ−2),α4=(g−2βgh2(Δx)22cos⁡⁡θ−22cos⁡⁡θ+4)(Δt8Δx(2sin⁡θ))I.

**Figure 2:**
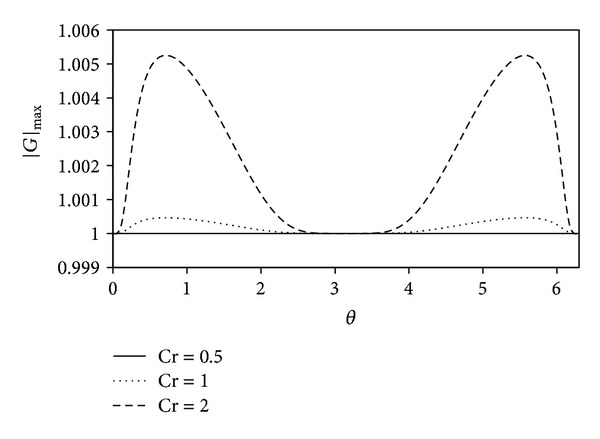
Largest modulus of the amplification factor |*G*|_max⁡_ in terms of phase angle *θ* for the corrector method.

The largest modulus of amplification factor in terms of phase angle *θ* is illustrated in Figure 2. 

Based on this figure, it is obvious that the corrector scheme is stable when the Courant number is less than or equal to 0.5.

## 4. Numerical Experiments 

Accuracy of the suggested model is examined by different simple and complex test cases. The first test considered is the regular wave shoaling on a constant slope beach where nonlinearity effect is examined. Time histories of the free surface elevation computed by the proposed model are compared against the existing results of experimental test conducted to predict the nonlinearity properties of the model. In continuation, in a more complex case, a time series of velocity in addition to free surface evaluation are also determined and compared with the available numerical and experimental results. Another test case considered which is more related to the dispersion property of the model is the simulation of periodic wave propagation over a bar. Here, free surface elevation determined numerically is compared against the experimental profile. Finally, the phenomenon of nonlinear wave propagation over a barred beach is modeled by the current numerical schemes. As a result, the wave decomposition is examined behind or beyond a bar, and the computed dispersed waves are compared against available numerical findings.

### 4.1. Regular Wave Shoaling on Constant Slope Beach

**Figure 3:**
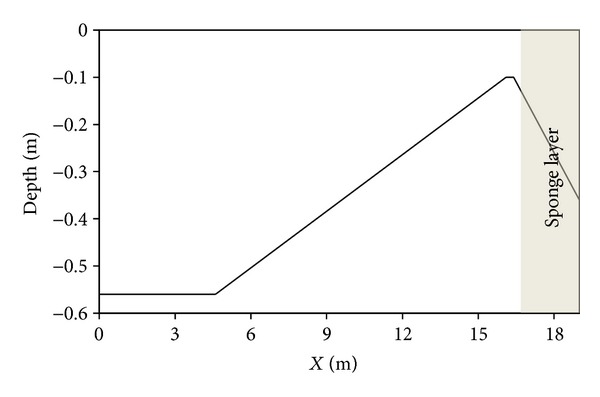
Schematic of the computational domain for regular wave shoaling on a constant slope beach.

A beach with a constant slope is the simplest assumption that can be considered for the morphological purpose. To demonstrate the strength of the present numerical scheme in modeling the nonlinear terms in the system of BNBE, the model is applied to simulate the propagation of regular wave over a constant slope beach where nonlinearity effect increases (under the effect of the slopes). Computational domain is shown in Figure 3.

The computational domain with length *L* = 19 m is divided into 489 elements. The slope is 1 : 25, and the starting point of the slope with respect to the inflow boundary is located at *x* = 4.6 m. Water depth at the inflow boundary is equal to 0.056 m and the regular wave is absorbed at the outflow boundary where the water depth is reduced to 0.1 m. The considered wave period and wavelength are 1.0 s and 1.55 m, respectively. The wave is allowed to propagate until the time *t* = 30.0 s, with a time step Δ*t* = 0.025 s. The velocity boundary condition at the incoming boundary for the simulation of regular wave is known from the linear wave theory.

**Figure 4:**
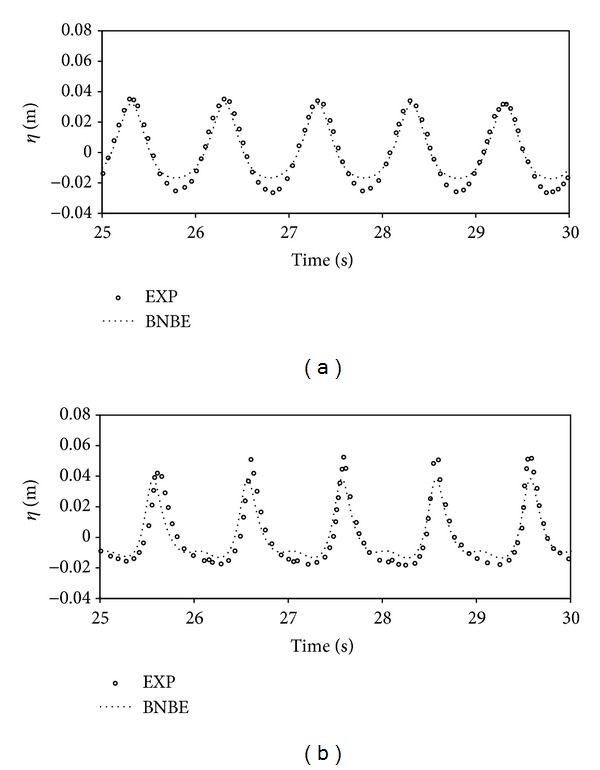
Comparison of time series of regular wave shoaling on a constant slope beach: *h* at (a) 0.24 m and (b) 0.1 m.

In order to analyze the accuracy of the obtained results by the current numerical scheme, time histories of the free surface level at the first and second gages, which are located at *h* = 0.24 m and at *h* = 0.1 m, are determined. The synchronized results of the numerical method and the available experimental data [5] are presented in Figure 4 for the sake of comparison. 

In both of the presented plots, the time histories of water surface elevation, computed at the first and second gages, using the suggested model, are in favorable agreement with the experimental data. It is quite clear that the presented numerical model has good ability to model the wave nonlinearity effects in the shoaling phenomena. 

### 4.2. Long Periodic Wave Propagation over Sloping Beaches

**Figure 5:**
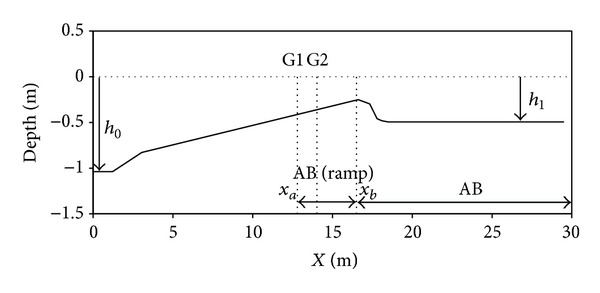
Schematic of the computational domain for 1D-BNBE computations of a periodic wave deep with height *H*_0_ and period *T*.

In this case study, the ability of the present model for determining the horizontal particle velocity near the bed and the nonlinearity effect of the free surface are investigated. Good prediction of horizontal particle velocity may be important when the cross-shore sediment transport in nearshore is to be modeled, if the Boussinesq equation is to be applied. Gilbert et al. [22], in continuation of the research done by Grilli et al. [23], studied the propagation of the long periodic waves over semiburied cylindrical objects at the bottom of a wave tank. The aim of that study was to find a numerical model for simulation of nonlinear wave forced sediment transport, where near bed flow increases near the objects in shallow water coastal area. For this situation, they offered an experimental as well as a numerical work. Their experiment involved a laboratory wave tank with a sandy bottom, while their numerical model included solution of a two-dimensional fully nonlinear potential flow (FNPF). Some experiments were run without any bottom obstacles. Here, these experimental cases were conducted to calculate the free surface and the particle velocity by one-dimensional BNBE model at definite gages for the case where there was no obstacle at the bottom. It was shown that the effect of the barrier is quite low indicating a maximum of 6% error in the water surface level [23]. Schematic of their numerical wave tank is illustrated in Figure 5. Wave is generated at the left boundary (*x* = 0) at the initial water depth of *h*_0_ = 1.05 m. Bed slope starts with a 1 : 6 slope at *x* = 1.219 m and is followed by a 1 : 24 slope at *x* = 3.048 m. 

As detailed in Figure 5, the surf zone is modeled by AB following the AB (ramp) which itself starts at *x*_*a*_ = 12.8 m in the AB. The seabed slope diminishes at the point *x*_*b*_ = 16.5 m, prior to the wave breaking point, and then (for *x* > *x*_*b*_) the water depth gradually increases to *h*_1_ = 0.5 m and rapidly ripples under the wave action. Experimental measurements are only made when the seabed shape levels out. The fully nonlinear potential flow model did not offer the precise shape of the ripples, as this would not strongly influence the shoaling waves [22, 23]. 

To dissipate the energy in order to prevent wave overturning, wave energy was absorbed for *x* > *x*_*b*_ in the present model and also in the FNPF model. 

**Figure 6:**
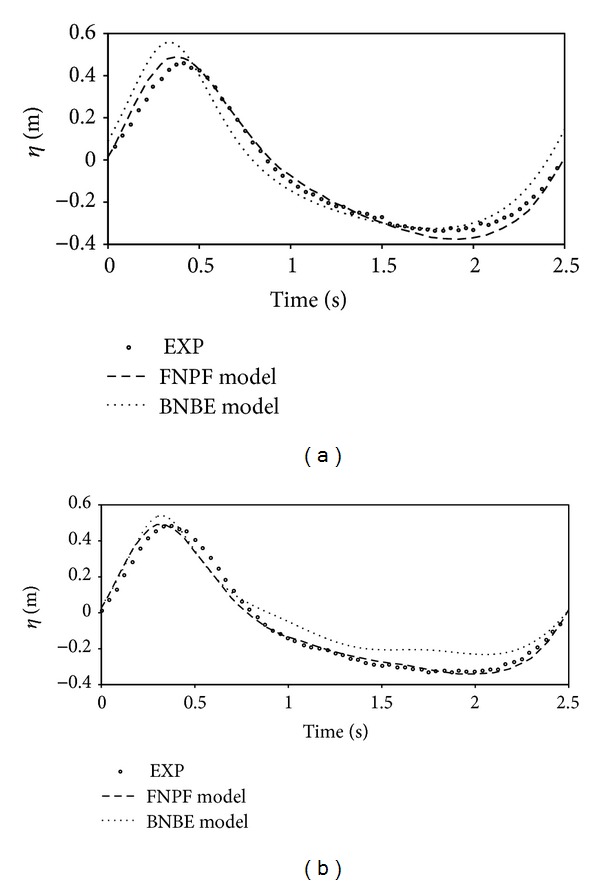
Comparison of the results for free surface elevations by BNBE model against the existing results of FNPF model and the experimental data at gages: (a) G1; (b) G2.

**Figure 7:**
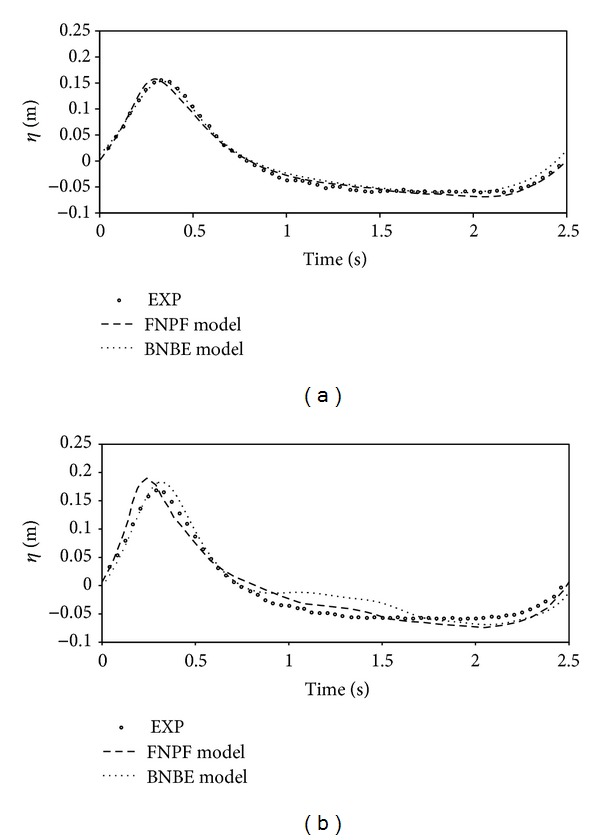
Comparison of the results for horizontal velocity by BNBE model against the existing results of FNPF model and the experimental data at gages: (a) G1; (b) G2.

Similar to the experimental test, a periodic wave with period *T* = 2*π*/*ω* = 2.5 s and height *H*_0_ = 0.19 m is simulated by the present model. Computational domain (shown in Figure 5) is discretized by 400 linear finite elements, and each time step is equal to Δ*t* = 0.025 s. Free surface elevations obtained by BNBE model are compared with the available experimental data and FNPF model at gages G1 (*x* = 12.802 m) and G2 (*x* = 14.021 m) as shown in Figure 6. Here, consistent trends are displayed by model at both gages. The agreement is quite good for the results at gage G1, but the computed result shows a little less overestimation at G2. On the other hand, horizontal velocity was calculated by BNBE model and compared with the available experimental data and the results of FNPF model. Horizontal velocity data was measured at 0.1 m above the bottom at gages G1 and G2 without the obstacle. As stated earlier, BNBE model computes the depth-integrated horizontal velocity.  Figure 7 shows these comparisons. The results display overestimation of the maximum velocity magnitude by 25% at all gages compared to FNPF model and experimental data. The reason is obvious. The velocity near the bed is smaller than the averaged velocity that is predicted by the Boussinesq models. Also, the asymmetry of the horizontal particle velocity calculated by the boussinesq models, compared to that found by the FNPF model and the experimental data at these gages, is relatively good. 

Comparison of the results in Figures 6 and 7 indicates lager onshore velocities under crests and smaller offshore velocities under troughs. Such velocity patterns are directly related to the observed sediment transport [24]. The good agreement of the velocity displayed can be indicative of the fact that BNBE can be used to determine the cross-shore sediment which will be used in future work.

### 4.3. Periodic Wave Propagation over a Bar

**Figure 8:**
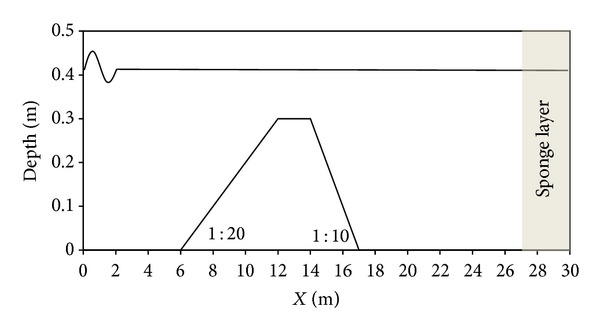
Bathymetry for the bar.

Before applying the suggested model for simulation of the wave propagation over the barred beach, a simple bar over a constant bed is considered here with the hope that wave properties (wave height and phase celerity) can be modeled accurately after the passing of waves over the bars. This test is more important for showing the accurate numerical modeling of the dispersion properties of the models. The experimental data of this test case was firstly published by Beji and Battjes [25].  Figure 8 illustrates the computational domain for modeling the flume experiment.

**Figure 9:**
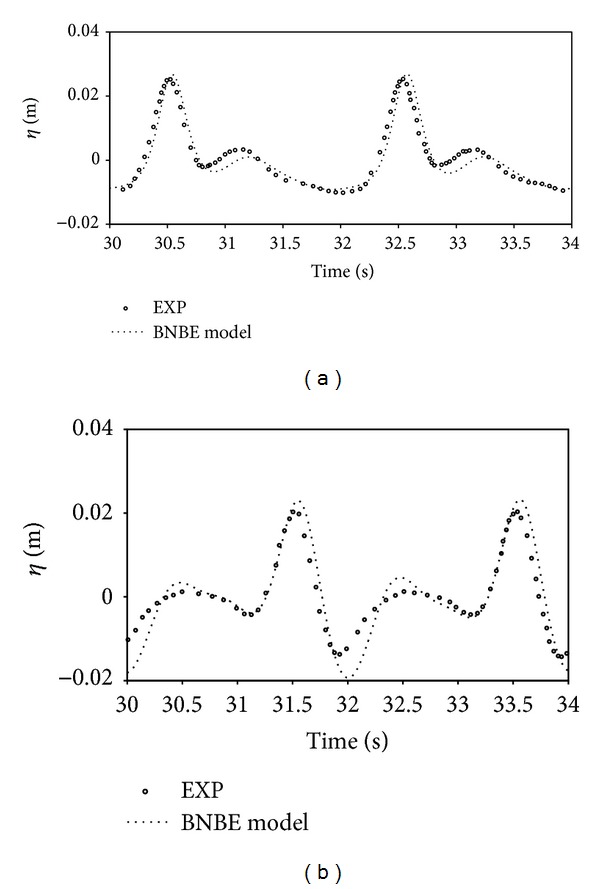
Comparison of time series of free surface over a bar, (a) *x* = 13.5 m; (b) *x* = 17.3 m.

The input wave is of wavelength *λ* = 3.73 m, wave period *T* = 2.02 s, and height *H* = 0.02 m. wave enters into a calm water of depth *h* = 0.40 m before reaching the bar. To neutralize the effect of exiting wave, a sponge layer is utilized at the right boundary. The computational domain is discretized with grid size elements of Δ*x* = 0.05 m in the wave propagation direction. The wave is allowed to propagate until the time *t* = 40.0 s, and a time step Δ*t* = 0.026 s is used for the time integration. Comparison of the numerical free surface elevation with the available experimental data for the gage stations at *x* = 13.5 and 17.3 m is shown in Figure 9. 

Results show no significant difference between the current numerical method and that of existing experimental data, and the model has the same behavior after passing the gage located at *x* = 17.3 m. 

### 4.4. Shoaling of Regular Waves over Barred Beaches

**Figure 10:**
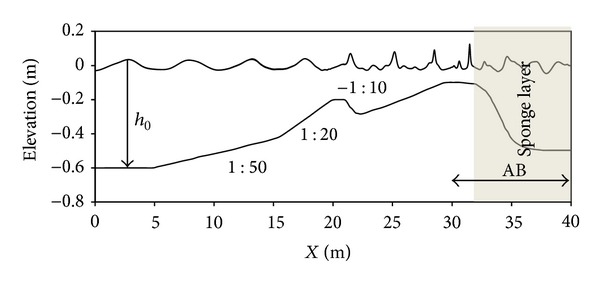
Schematic of the computational domain for a periodic wave modeling.

The assumption of a constant slope beach is often not a good approximation as sandy coasts exposed to a wave climate will often build up barred beaches [1]. Schematic of this numerical test which was first used by Grilli and Horrillo (1999) is shown in Figure 10. 

**Figure 11:**
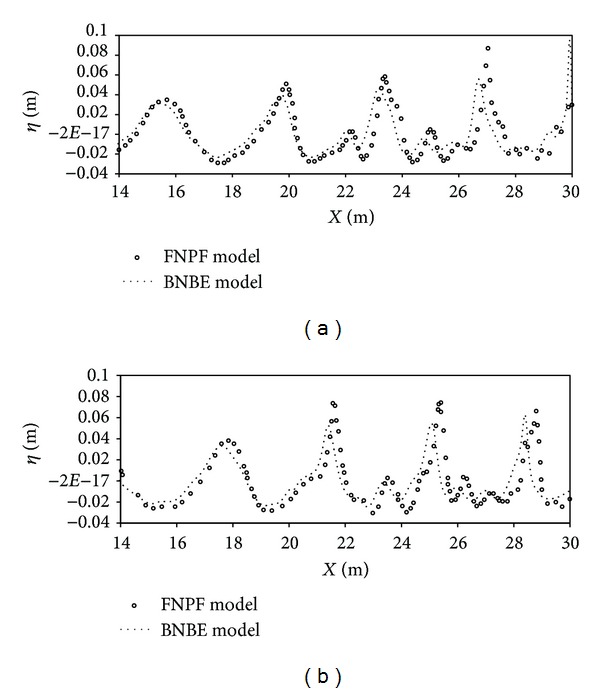
Computed surface elevation for wave period *T* = 2.394 s: (a) *t* = 3.592*T* s and (b) *t* = 3.911*T* s.

The main profile considered for the beach has the same form as Dean's [26] equilibrium beach profile. This profile has an average slope of 1 : 50 along with a bar of 0.2 m height with a 1 : 20 seaward slope and 1 : 10 shoreward slope located toward its top. Geometrical profile of this bar is similar to that used by Beji and Battjes in their experiments [25]. Similar to their test cases, wave in the current numerical study is decomposed after propagating over the bar. After passing the bar, the wave nonlinearity increases until the wave reaches the breaking zone (i.e., AB). A sponge layer is located at this zone to absorb the outgoing wave. The length of the domain is *L* = 40.0 m and is divided into 1000 linear elements along the *x* axis. The constant depth in the primary part of domain is equal to 0.6 m. A regular wave with a height of 0.06 m is generated at the incoming boundary. In the first test conducted here, a regular wave with period *T* = 2.394 s is simulated. Free surface elevation computed by the present numerical model for *t* = 3.592*T* s and *t* = 3.911*T* s is compared against the existing fully nonlinear potential flow model (FNPF) [27] in Figure 11. 

**Figure 12:**
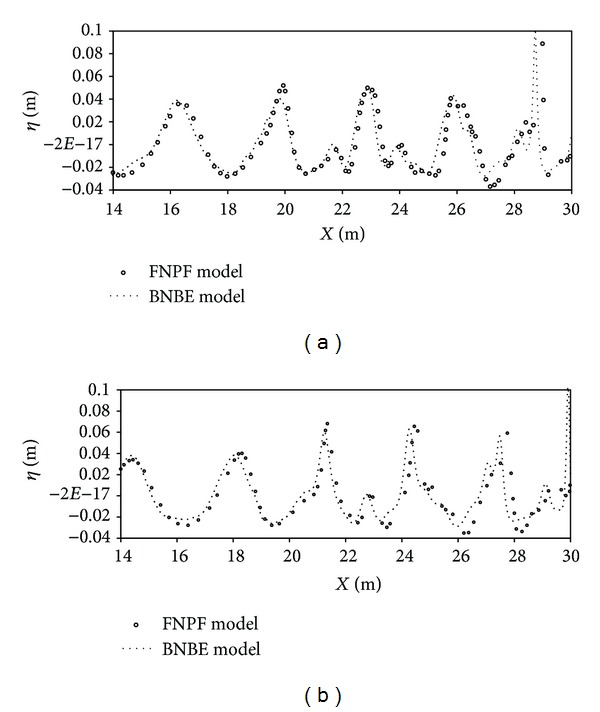
Computed surface elevation for wave period *T* = 2.075 s: (a) *t* = 12.5*T* s and (b) *t* = 13.0*T* s.

In the second test, numerical results and the existing FNPF results at *t* = 12.5*T* s and *t* = 13.0*T* s are presented in Figure 12 with smaller wave period of *T* = 2.075 s. 

Comparison of the results produced by the present models with that of the existing numerical results obtained by the FNPF model indicates that the nonlinearity and dispersive properties of the wave are predicted well. Figure 12 shows that waves height predicted by BNBE model is underestimated just like the results obtained in the first test case. For *x* > 26, the wave nonlinearity increases after passing over the slope, and consequently a slight phase difference is observed between the results of the present model and that of FNPF. Strong nonlinear wave decomposition in the region beyond the bar is predicted by BNBE which is similar to the results of the FNPF model. However, as the wave reaches the top of the slope, that is, the breaking zone, the nonlinearity increases and becomes stronger, but the wave height is overpredicted by the BNBE model (as shown in Figure 12(b)). Considering the fact that BNBE model is a one-dimensional approximation, the obtained numerical results may be considered remarkable. 

## 5. Conclusions

In the current study, a particular numerical method is presented for the Beji and Nadaoka extended Boussinesq equations to determine the local wave properties (i.e., wave height and wave velocity) in nearshore zone by one-dimensional cases. Numerical forms of these equations are revisited, and, by introducing a novel form of the auxiliary variable, equations are rewritten in lower-order forms making the application of the linear finite element method possible. 

In order to show how well the nonlinearity and dispersion properties of the waves are predicated, two benchmark tests have been conducted. Regular wave shoaling on a constant slope beach and periodic wave propagation over a bar have been modeled. In these test cases, accurate modeling of nonlinearity and dispersion of regular waves are of high importance, respectively. Results of these numerical case studies show good agreements for numerical model against the available experimental findings. In the case of long periodic wave propagation over the sloped beach, the nonlinearity of the wave was noticed to increase while reaching the top of the slope. However, even though the asymmetric nature of wave shape was not predicated well by Beji and Nadaoka's model, the particles' velocities were well predicted, compared against the experimental results. This is an indicative of the fact that the Beji and Nadaoka Boussinesq equations can be developed for computation of the cross-shore sediment transport under the influence of long periodic wave near the shore.

Modeling the periodic waves over the barred beaches was studied in another test, and the obtained results were compared with the numerical results available by the FNPF model. Comparison showed that the Beji and Nadaoka Boussinesq equations are almost capable to model the decomposition of the nonlinear waves beyond the bars. Contrary to the two-dimensional FNPF model, this was achieved only by a one-dimensional approximation.

Based on the results presented, it can be concluded that the current numerical scheme can be further developed to simulate any morphological development of coastal profiles under the influence of long periodic waves.
